# Discovering conditional co-regulated protein complexes by integrating diverse data sources

**DOI:** 10.1186/1752-0509-4-S2-S4

**Published:** 2010-09-13

**Authors:** Fei Luo, Juan Liu, Jinyan Li

**Affiliations:** 1 School of Computer, Wuhan University, Wuhan, Hubei, China; 2 School of Computer Engineering, Nanyang Technological University, Singapore

## Abstract

**Abstract:**

**Background**

Proteins interacting with each other as a complex play an important role in many molecular processes and functions. Directly detecting protein complexes is still costly, whereas many protein-protein interaction (PPI) maps for model organisms are available owing to the fast development of high-throughput PPI detecting techniques. These binary PPI data provides fundamental and abundant information for inferring new protein complexes. However, PPI data from different experiments do not overlap very much usually. The main reason is that the functions of proteins can activate only on certain environment or stimulus. In a short, PPI is condition-specific. Therefore specifying the conditions on when complexes are present is necessary for a deep understanding of their behaviours. Meanwhile, proteins have various interaction ways and control mechanisms to form different kinds of complexes. Thus the discovery of a certain type of complexes should depend on their own distinct biological or topological characteristics. We do not attempt to find all kinds of complexes by using certain features. Here, we integrate transcription regulation data (TR), gene expression data (GE) and protein-protein interaction data at the systems biology level to discover a special kind of protein complex called conditional co-regulated protein complexes. A conditional co-regulated protein complex has three remarkable features: the coding genes of the member proteins share the same transcription factor (TF), under a certain condition the coding genes express co-ordinately and the member proteins interact mutually as a complex to implement a common biological function.

**Results**

A framework of discovering the conditional co-regulated protein complexes is proposed. Testing on the Yeast data sets under the Cell Cycle, DNA Damage and Dauxic Shift conditions, we identified a total of 29 conditional co-regulated complexes, among which the coding genes in 14 complexes show a strong association with their TFs activity. Based on the close relationship among co-regulation, co-expression and protein-protein interactions in the conditional co-regulated protein complexes, 39 novel TRs were predicted and explained.

**Conclusions**

This paper was initiated to study conditional co-regulated protein complexes by integrating multiple data sources. Taking into consideration the influence of TFs activity on the protein interactions, we found that the expression coherence of the protein complexes’ coding genes changed in accordance to their TFs’ activity, which implied that the proteins’ interactions also changed in response to the environments. Based on the three features of conditional co-regulated protein complexes, new transcriptional regulation interactions were predicted.

## Background 

Protein complexes perform all kinds of fundamental biological functions in cells. Thus far, there have few reliable techniques to directly detect protein complexes in a large-scale style, whereas binary interaction between two proteins is relatively easy to be detected by experiments such as Yeast Two-Hybrid (Y2H) [1], tandem affinity purification (TAP) [2] and Mass Spectrometry (MS) [3]. Many protein-protein interaction (PPI) networks of model organisms such as yeast, fruit fly and so on have been mapped. They provide fundamental and abundant data for computational approaches to the inference of new type protein complexes. However, it has been reported that protein interaction data produced by different experiments for the same organism are often associated with high false positive and false negative rates which lead to a low overlapping degree between their results [4]. For example, the common PPI between the two different mass-spectrometry approaches stands at 1,728 pairs, which correspond to only 27.5% of PPI detected by TAP or only 19.2% of PPI detected by high-throughput mass-spectrometric protein complex identification [5]. It’s partly due to the limitations of the associated experimental techniques, and the more to point is the dynamic nature of the protein interaction maps. Currently, most of computational approaches mainly use large-scale statistically oriented study or exact local topological analysis of protein complexes. The former ones could acquire the information about the global structural features including particular degree distribution [6], clustering properties [7] and possible hierarchical structure of the examined networks [8] and the later ones were focused on the discovery of functional motifs [9], themes [10], and modules [11]. Recently, a few of works [12-16] tried to answer the question when the complexes present and how to use for other applications. In the point of biology view, interactions between biological molecules including protein–protein interactions are dynamically regulated both in time and in space. Individual proteins can participate in the formation of a variety of different protein complexes and protein complexes have different degrees of stability over conditions. In order to understand the behaviours and functions of protein complexes precisely, it’s necessary to take the condition-specific features into account. 

Meanwhile, different types of protein complexes have their own distinct biological ‘pattern’ or ‘topology’ characteristics. Taking the topological feature of complexes as example, some complexes can be modelled as ‘clique’ whose members are densely connected within themselves but sparsely connected with the rest of the network [17,18], while other ones can be modelled as ‘star’ where there is a ‘hub’ unit playing a central functional role connected to its neighbours [19,20]. Thus, the discovery of certain kind of protein complexes strongly depends on their definition according to their own distinct characteristics. Recently, Jansen [21] found that subunits of the same protein complex showed significant co-expression, both in terms of similarities of absolute mRNA levels and expression profiles. Nitin [22] studied the correlation between gene expression profiles and protein-protein interaction on four evolutionarily diverse species: human, mouse, yeast and E Coli. They found that the gene expression profiles of protein-protein interacting pairs were highly correlated in E.Coli and the likelihood of predicting protein interactions from highly correlated expression data was increased by using additional protocol for other three species. Zhang [10] observed an outstanding phenomenon that co-regulated coding genes with similar profiles often lead to intensive interactions between their protein products and forming a protein complex. Tan[23] proposed an innovative concept of co-regulated protein complex where proteins were encoded by genes that are regulated by the same transcription factors (TFs). These interesting results imply that there is a tight linkage between transcription regulation, gene expression and protein-protein interaction.

Instead of defining protein complexes only by their topological characteristics, we make use of the three remarkable features of conditional co-regulated protein complexes: (1) the coding genes of the member proteins share the same active transcription factor, (2) the coding genes express co-ordinately and (3) the member proteins mutually interact as a complex to implement a common biological function. In order to study their associations under some given condition, we integrate transcription regulation data (**TR**), gene expression data (**GE**) and protein-protein interaction data (**PPI**) at the level of systems biology. Furthermore, we consider the condition-specific features of the interactions including **TR** and **PPI**. Because accurate temporal parameters are not yet available for many protein–protein interactions, a common way to estimate temporal characteristics of protein products is using compilations of GE data [24]. We first use gene expression level in GE as the criterion to judge the activity of TFs in the TR. Then starting with the active TFs, conditional co-regulated complex seeds are identified in the PPI network. Finally, extra members of complexes are found by extensive searching, during which new transcription regulation interactions are predicted.

## Methods

**Figure 1:**
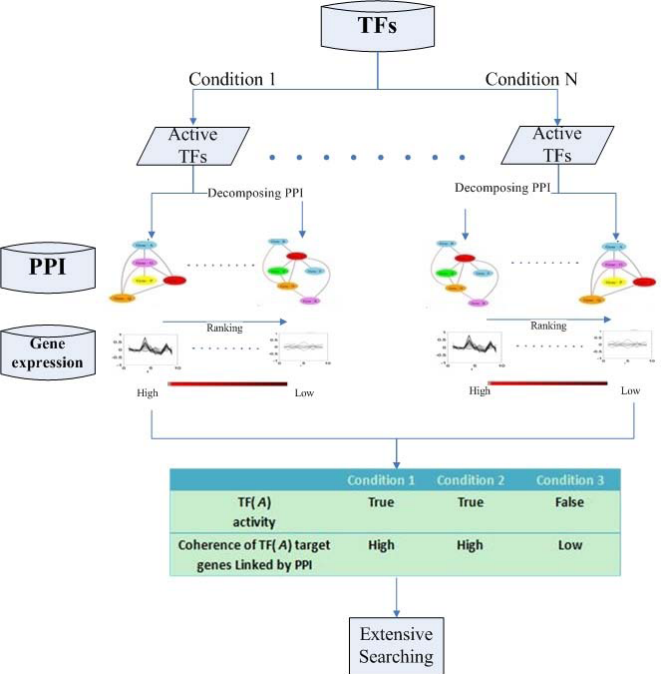
**Schematic overview of conditional co-regulated protein complex identification.** Given a condition, the active TFs are identified first and find out all target genes from **TR** data for each active TF and their protein products. The proteins and protein-protein interaction form a sub-network in the global **PPI** network. This sub-network will be decomposed into several weakly connected components (WCCs). A greedy searching way would be implemented to find out possible candidate groups, which is a linked sub-network in the WCC. We calculate a coherent score for candidates. The higher the score is, the more likely the candidate is to become a co-regulated complex. Finally, a relationship between the activity of TF and coherence of candidates with the conditions’ changing is investigated, which can be used to judge TF impact on their products’ function. Candidates with high coherent scores and their nodes exceeding a threshold are used as seeds to search additional extra members and predict new TRs.

The genetic information of biological systems contained in genes is first initiated by transcriptional factors, and then mRNAs are translated to proteins to execute biological functions. The activity of TFs could make an impact on their downstream products' the functions. Accordingly, we propose the framework to discover conditional co-regulated protein complex as follows. The framework is shown in the Figure 1.

### Preliminary definitions

Let ***T*** = {*tf*_1_,*tf*_2_,…*tf_s_*} be a set of transcription factors, ***P*** = {*p*_1_,*p*_2_,…*p_m_*} be a set of proteins, and ***G*** = **{*x***_1_,***x***_2_,…,***x**_n_***}** be a ***GE*** data set. ***PPI*** network is represented by *PPI*=(***P***, ***E**_PPI_*) , where ***E**_PPI_* = {(*p_i_*, *p_j_*) | *p_i_*, *p_j_* ∊ ***P***}. ***TR*** interaction data is denoted by *TI* = (***T***, ***E**_TI_*) , where ***E**_TI_* = {(*tf_i_*, ***x**_j_*) | *tf_i_* ∊ ***T***, ***x**_j_* ∊ ***G***}. In particular, ***x_for p_*** stands for the coding gene for protein *p*. 

Given a ***GE*** under certain condition, *L*(*P*', *E_PPI_*') ⊆ *PPI* is a conditional protein complex, if and only if it meets the following requirements. 

(i) All ***x_for p_*** where *p* ∊ *P* ', share the same active *tf* ∊ *T* under the given condition,

(ii) *L* is a connected graph,

(iii) The coherent score *Score*(*L*) of *L* is greater than the threshold *α* and its score degree is consistent with the activity of its TF as least *θ* conditions in all conditions.

The above three requirements correspond to the three features of co-regulated protein complexes. Parameters *α* and *θ* in (iii) are thresholds used to distinguish a conditional co-regulated protein complex from the others.

### Identification of active TFs

Identifying the active TFs under given conditions is challenging. A recent research work [25] adopted the assumption that the regulators are themselves transcriptionally regulated. Therefore, their expression profiles can provide informative clues to indicate their activity level. TFs are identified as being ‘active’ at certain condition if they reach sufficiently high expression levels. We use the **Trace-Back** algorithm [26] to identify the active TFs when conditions are given.

### Identification of co-regulated complex seeds

For all target genes of each active TF, their protein products and their protein-protein interactions form a local sub-network in the global PPI network. This sub-network will be decomposed into several weakly connected components (WCCs). Because WCCs disjoint each other, one TF may correspond to more than one WCC. We take a **core-neighbour** strategy to search our target complexes.  The procedure includes two stages: the first one is to search the core part (also called **seed**) in the WCCs in a greedy way and the second one is to conduct an extensive search for extra members of the core in the global PPI in a heuristic way. The computational benefit is obvious as compared to ten thousands of edges in a global PPI, the search space in WCCs decreases by orders of magnitude. As constrained by the requirement (iii) in the definition of conditional protein complexes, we also use a coherent scoring threshold α to judge whether a group is a seed. The arc to vertex number of these WCCs varies greatly from several ones to hundreds. It’s infeasible to find out all combinations of the proteins which are linked by hundreds of interactions in the WCC due to the computational cost. However, in practice the member proteins in most known protein complexes do not exceed ten. Therefore, we set two parameters *λ* and *β* to limit the minimum and maximum edges to narrow down the search space. We take a greedy method to search all possible groups meeting the two parameters in WCCs, and then we measure their coherent score in the given condition, and finally identify seeds whose score exceeds *α* and its score degree is consistent with the activity of its TF as least *θ* conditions.

### Coherence measurement

As the coding genes in a co-regulated complex have coherent expression, the change in the coherence degree can indicate different states of the complex function. Taking the scoring methods as used in [12,16], a protein group is denoted by *L* = (*V*, *E*). For any *v_i_*, *v_j_* ∊*V*, if  *e* = (*v_i_*, *v_j_*) ∊ *E*, we calculate a score of the coherence between *v_i_*, *v_j_* by

              (1)

Where *Corr *(*v_i_*, *v_j_*) is the Pearson Correlation Coefficient between the coding genes of protein* i* and protein* j* to reflect their coherence. Different from the formula used in [16], we do not include the individual gene’s expression variation measured by std () for two reasons. The first reason is that some genes could be active with low expression variation, and the second reason is that the score could be still high with very high expression variation and relatively low coherence.

Thus, the coherence score of *L*, denoted by *T*(*L*), is the sum over the scores of all the edges in *L*:

                        (2)

We note that the coherence score can be influenced by the number of edges in *L*. Guo[16] and Ideke [13] have proved that the problem could be solved in the following way. In order to compare the coherence between groups with different number of edges, for *L* with *K* edges, we randomly choose 10 000 sub-graphs with *K* edges from the PPI network and compute their score by using formula (2), then calculate the average and standard deviation value of these 10 000 graphs and use formula (3) to standardize the final score for seed *L* with *K* edges. After standardization, groups with different number of edges can be compared with the coherence.

                     (3)

### Extensive search

**Figure 2:**
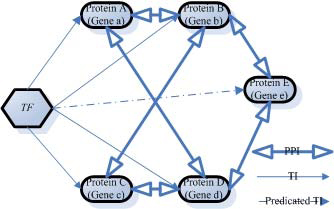
**Inferring new TR interactions.** From the existing **TR** interaction data, genes *a*, *b*, *c*, and *d* are known to be target genes of a TF, and *A*, *B*, *C*, and *D* stands for their proteins. From the **PPI** network and **GE**, if we observe that the proteins *A*, *B*, *C*, *D* and an additional protein *E* interact intensively one another, and that their coding genes *a*, *b*, *c*, *d* and *e* express co-ordinately, we can predict that the TF also regulate *e* under the same condition. The reason is that *E* is so similar to the co-regulated protein group of *A*, *B*, *C*, *D* at the levels gene expression and protein-protein interaction that it can be inferred that *e* also has **TR** interactions associated with TF just as *a*, *b*, *c* and *d* do, although the known** TR** dataset does not indicate this.

In the global **PPI** network, we seek extra proteins of the seeds which interact intensively and co-express with the given seed with a high coherence score. A protein which expresses differently with the seed will make the score decrease; while a protein which expresses consistently with most parts of the seed will increase the score. Therefore, the search process can be converted to optimize the score by adjusting the structure of the sub-graph starting from the seed. Our extensive searching implements a simulated annealing procedure for every seed. The proteins that **TR** does not indicate the same TF with the seed can be added into the seed by the extensive search. In this situation we can predict a new **TR** interaction according to the inference model shown in the Figure 2. The pseudo code is shown in Table 1, where *L_initial_* is corresponding to the seed. The input parameters *T_start_*, *T_end_* are the initial and ending temperature respectively, and *N* is the iteration number.

**Table 1:** Pseudo codes of our search method

Input:	*L_initial_, T_start_, T_end_, N*
Output:	*L_rs_*
step1:	*L_rs_ = L_initial_*, calculates *Score* (*L _rs_*)
step2:	for *i* = 1 to *N*
step2.1:	Calculates
step2.2:	*L_try_* = *L_rs_*
step2.3	randomly choose a vertex *v* from *L_try_* , and choose a random arch *e* from *E _PPI_* , one of whose vertex is *v.*
step2.4:	if (*e* ∊ *L_try_*) if(*L_try_* is still connected without *e* and *e* ∉ *L*_in_*_itial_*) delete *e* from *L_try_* else add *e* into* L_try_*
step2.5:	Calculates *Score*(*L_try_*)
step2.6:	Δ = *Score* (L*_try_*) − *Score* (L*_rs_*)
step2.7:	If(Δ > 0) * L_rs_* = *L_try_* else *L_rs_* = *L_try_* with the probability
step3:	end

## Results 

### Data collection

**Figure 3:**
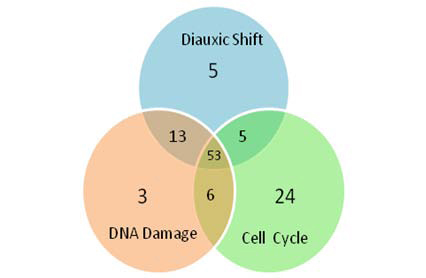
**Distribution of active TFs over the three conditions.** In the total 142 TFs, 88, 76 and 75 active TFs are determined in Cell Cycle, Diauxic Shift and DNA Damaging conditions respectively.

The Yeast dataset used in the method evaluation involves three biological conditions: Cell Cycle [27], DNA Damaging [28], Diauxic Shift [29]. The Cell Cycle wet-lab experiment includes expression measurements of 6 178 genes measured at 77 time points. The DNA Damaging experiment has 6 129 genes’ expression values with 52 sampling points. The Diauxic Shift dataset consists of 6 068 gene expression profiles with 7 time points. We also use the total 7 074 **TRs** data from Luscombe’s work [26], which uses the **Trace-Back** algorithm to determine active TFs. The distribution of active TFs in the three conditions is shown in Figure 3. Total 54 015 protein-protein interactions from the interoporc [30] are also used in our work. In the data pre-processing, we directly neglected the time point with missing value when calculating the correlation coefficient. Because there are three different molecular types of data in our work, we unified data symbols by mapping all symbols into gene ID as the standard reference. If the corresponding coding genes of the proteins in the PPI cannot be found in the GE dataset over all conditions, we excluded those proteins from the PPI data. The data used in this work are listed as Additional file 1, Additional file 2, and Additional file 3.

Starting from the active TFs, all WCCs are decomposed according to the structure of the global PPI network. The edge number of the WCCs varies greatly. The maximal edge number of the WWC for the transcription factor YKL112W can reach 293. We set *λ* = 2 and *β* = 21 to greedily search complex seeds from the all possible parts whose edge number is between *λ* and *β* in the WCCs. If two candidates share common 80% proteins, we take the one with a higher coherent score. Because there is no gold-standard threshold for the coherent score to judge which seeds can be in a co-regulated complex, we had to take the top ones in the ranking list to guarantee the prediction accuracy with the parameter *α* = 1.90.

**Figure 4:**
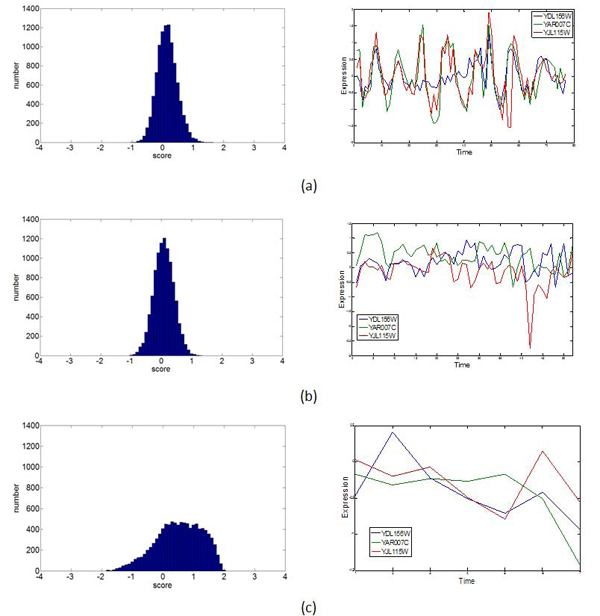
**The distribution histogram.** T (*L*_2edges_random_) in 10000 random sampling and the corresponding expression profiles of the complex seed consisting of YDL156W, YAR007C, and YJL115W with 2 PPI edges. (a) Cell Cycle, (b) DNA damaging，(c) Diauxic Shift. In the left panels, the X axis is the T(***L***) score and each interval is 0.1. Y axis is the frequency of the T (***L***) in each interval. The right panels are the expression profiles YDL156W, YAR007C, and YJL115W under corresponding conditions.

Table 2 lists a total of 29 complex seeds (***YNL216W*** and*** YPR104C*** regulated the common seed) identified by our method from 21 TFs who’s at least active in one of the three conditions，and all seeds’ coherence degree is consistent with activity of their TF at least *θ* = 2. In Table 2, c1 represents cell cycle, c2 stands for DNA Damaging and c3 denotes Diauxic Shift. From this table, we can see that the coherence degree of the coding genes in the seeds corresponding to the TFs ***YOR028C, YDR451C, YLR183C, YBL021C, YGL013C, YDL020C, YDR207C, YKL112W, YCR065W*** and*** YKL109W*** are perfectly consistent with their TFs’ activity. As the expression coherent levels of the proteins’ coding genes change in accordance to the activities of the TFs in all conditions, we can infer that the functions of their protein complexes also follow the pace with their coding genes and TFs. They are perfect conditional co-regulated protein complex seeds. Take the complex seed ***L*** consisting of YDL156W, YAR007C, and YJL115W with 2 edges as example. Their transcription factor is YDR451C. Figure 4 depicts the T(***L***_2edges_random_) distribution generated by 10 000 random sampling and the corresponding expression profiles of the complex seed ***L***. This complex seed has T(***L***)=0.6386, T(***L***)=0.0605, T(***L***)=0.3513 and Score(***L***)=3.18, Score(***L***)=-0.49, Score(***L***)=0.25 under the C1, C2 and C3 conditions respectively. Comparing between C2 and C3, T(***L***)=0.6386 in C1 is significant. The probability of T(***L***) over 0.63 is 0.07 in the distribution by random sampling as showed in left panel figure 4(a). Both T(***L***) and Score(***L***) score are consistent with the activity of their TF.

**Table 2:** conditional co-regulated complex seeds under three conditions

***TFs***	***Complex seed***	***Score(·)***			***MIPS ***	***TFs activity***		
		***C1***	***C2***	***C3***		***C1***	***C2***	***C3***

***YOR028C***	YHR047C,YHR128W ,***YJR145C , YNL178W***	0.13	1.93	1.92	500.40.20	F	T	T
YOL108C	***YKL182W*** ,YLR153C,YNR016C ***YPL231W***	0.71	3.17	2.08	170	T	T	T
***YDR451C***	***YDL156W,YAR007C***,YJL115W	3.18	-0.49	0.25	550.1.212	T	F	F
***YLR183C** ^1^*	YER159C,***YER148W,YBR198C***	1.90	0.82	0.80	550.1.196	T	F	F
YDR501W	***YHR148W***,YIL019W,***YER082C***	2.55	3.51	1.65	550.1.109	T	F	F
YEL009C	YOR108W,***YOL058W***,YNL104C	1.93	2.31	-0.4	550.2.327	T	T	T
***YLR183C** ^2^*	YKR070W,***YER012W***,YMR276W	2.74	1.12	0.06	360.10.10	T	F	F
***YBL021C***	***YPR191W,YOR065W,YHR001W-A,YJL166W***	2.42	3.07	1.92	420.3	T	T	T
YDL056W	***YDL003W,YIL026C,YJL074C***, YMR076C	4.48	1.29	2.08	475.05	T	T	T
YKL062W	***YIL177C,YBR126C,YDR074W***, YCL040W,YFR053C	2.55	2.37	-1.1	550.1.29	T	T	T
***YGL013C***	***YDL148C***,YER074W,YHR193C	2.03	2.08	1.91	310	T	T	T
YDL056W	***YNL312W,YDR097C,YAR007C***, YER095W,YER078C	5.21	1.95	-0.6	550.1.202	T	T	T
***YDL020C***	***YGL048C,YOR259C,YOR117W, YDL007W***	4.30	4.64	2.36	360.10.20	T	T	T
***YDR207C***	***YHR005C***,YFL026W,YLR452C	3.35	0.56	-0.1	470.30.10	T	F	F
YML007W	YGR209C,YLR043C,YLR109W, ***YML028W***	3.04	2.36	0.58	550.1.41	T	T	T
YKL112W	***YBL038W***, YHR090C, ***YJL063C***, YLR399C, YNL306W, YNR037C	3.58	1.91	1.48	500.60.10	T	T	T
***YKL112W** ^1^*	YEL037C,***YHR200W,YJL008C***, ***YOR117W,YOR261C***	3.03	3.15	1.95	550.1.41	T	T	T
***YKL112W** ^2^*	YNL255C,YNR038W,***YOL077C, YOR206W,YKL014C,YKL172W, YKR081C,YLL034C***,YDL208W, YBL039C,YKL029C,YDR312W, YFL037W,YLR330W	7.84	5.22	4.54	550.1.149	T	T	T
***YLR183C** ^3^*	***YBL002W,YBL003C***,YER091C, YNL068C	3.05	1.35	1.00	320	T	F	F
YNL216W	YEL054C,***YFR031C-A,YIL148W, YML073C***,YOL086C	3.78	1.5	0	500.40.10	T	F	F
YPR104C	YEL054C,***YFR031CA,YIL148W, YML073C,***YOL086C	3.78	1.5	0	500.40.10	T	F	F
YER111C	***YDR224C,YDR225W***,YDR507C, YOL012C	3.26	2.38	1.89	320	T	F	F
YEL009C	***YER086W,YJR109C***,YLR355C	2.83	1.96	0.22	550.1.195	T	T	T
YGL073W	YAL005C,YLL024C,***YNL007C***, YPL240C,***YDR214W***,YLL026W, ***YLR216C***	11.1	5.75	-0.4	550.2.360	T	T	T
YBR049C	***YDR156W ,YJL148W ,YNL113W*** , YPL204W ,YPL231W	4.31	1.12	1.92	510.1	T	T	T
YBR049C	YDL213C,YGL120C,***YKL081W, YKL104C,YER086W***	2.75	0.88	1.91	550.1.103	T	T	T
***YKL109W***	YBL099W,YDR298C,***YDR529C,*** YJR121W,***YOR065W,YPR191W, YEL024W***	6.35	0.67	4.09	420.3	T	F	T
***YKL112W** ^3^*	***YKL060C,YKL144C,YOR210W, YPR110C,YPR187W,YOR207C, YOR116C***	3.03	2.79	1.91	550.1.213	T	T	T
***YCR065W***	YDL141W,YDR412W,***YNR054C, YPL217C***	3.76	1.91	1.03	550.1.125	T	T	F
YBR049C	***YIL148W,YMR121C***,YNL111C, YPR074C	3.49	1.25	2.57	500.40.10	T	T	T

In order to validate whether the proteins can form a complex, we identify the corresponding MIPS complexes which contain as many proteins in the predicted complex seeds as possible. 21/29 seeds have over 50% proteins covered by the corresponding MIPS complexes (the coverage genes are shown by bold). However, TFs are not the only factors to determine the behaviours of the coding genes. For example, we found that the complex seeds belonging to TF YBR049C may be influenced by other factors or stimulus, as YBR049C is active in DNA Damaging, but all its complex seeds show low coherent degree in DNA Damaging. We guess it is caused by DNA Damaging. In Table 2, some value is zero. It’s because the GE under the given condition does not cover the target genes. 

### Extensive search 

After the extensive search, not only the new TRs could be predicted, but also the extra members for the core seeds could be found. First to show whether the simulated annealing algorithm can reach a convergence point and to illustrate the parameter settings, we conducted an experiment to investigate how the score and edge number are changed with the iterations for the example complex seed ***L*** that consists of YDL156W, YAR007C, and YJL115W with 2 edges. Unfortunately, for the simulated annealing algorithm, there are no choices of parameters that will be good for all problems, and there is no general way to find the best choices for a given problem [31]. In theory, the final result could not be decided by the initial state and parameter setting, but the optimal parameter setting could have a significant impact on the method's effectiveness. 

**Figure 5:**
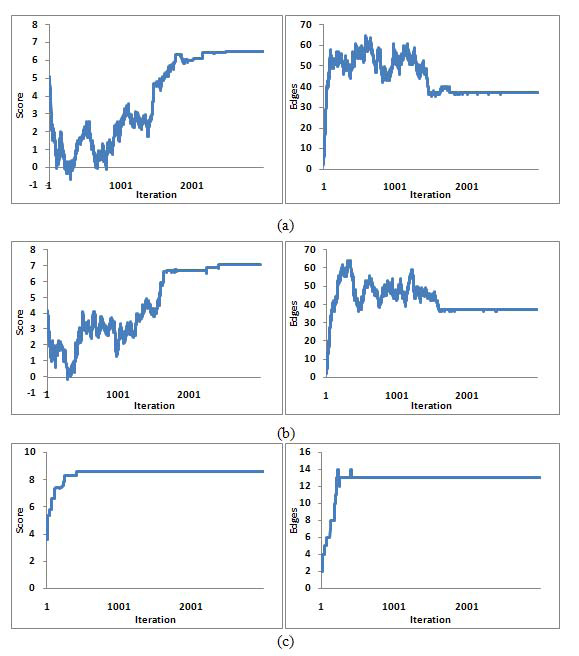
**The variation of Score(*L*) and edges during annealing process in extensive searching.** (a) *T*_start_=2, *T_e_*_nd_=0.01, *N*=3000, (b) *T*_start_=1, *T*_end_=0.01, *N*=3000, (c) *T*_start_=0.05, *T*_end_=0.01, *N*=3000

Three annealing runs starting from different initial annealing temperatures *T*_start_=2, *T*_start_=1 and *T*_start_=0.05 are shown in Figure 5. We could see that all of the results converge. When the distance of *T*_start_ and *T*_end_ is big, it will have big acceptance probability for the weak candidates. It could be observed in heading parts of the curve in (a) and (b), whose Score(*L*) decreased rapidly. When the temperature is cooling down, the weak candidates are likely excluded. However, it could jump out the local optimality to global optimality. In contrast, when *T*_start_ is near to *T*_end_, it’s more possible to reject the weak candidates and easily to reach the local optimal. The parameter *N* decides the searching times. If *N* is small, it couldn’t reach all possible searching space. Therefore, *N* should be set a bit big. Because our framework is based on **core-neighbour** strategy, the optimal local seeds have been identified and it could set *T*_start_ near to* T*_end_ to reject the weak candidates.

**Figure 6:**
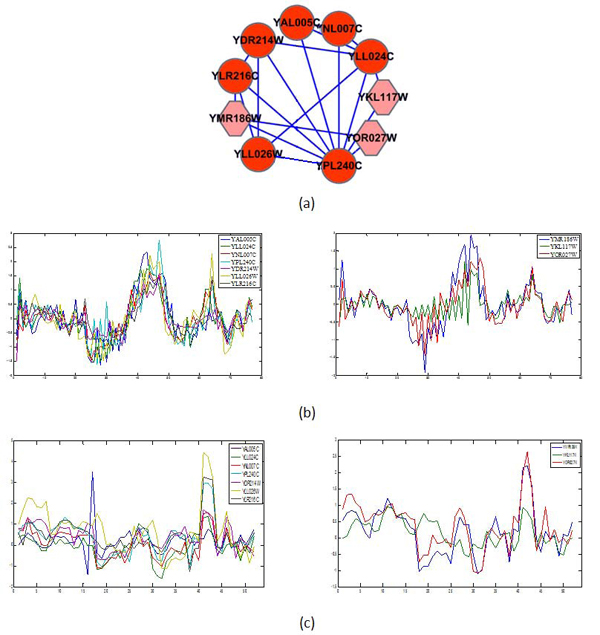
**The topology of seed and final result of Hsf1 (YGL073W).** The upper panel (a) is the topology of the seed and the final result of the extensive search. ’Hexagon’ is an added node in the final result and ’Circle’ is the node in the seed. The lower panel (b) and (c) shows the coding genes’ expression profiles of the proteins in the seed and the added proteins in the final result of Cell Cycle and DNA Damaging respectively.

The seed corresponding to the TF Hsf1 (YGL073W) had the highest score in the cell cycle (11.1) and DNA Damaging (5.75) conditions. As mentioned in the method part, we can use co-expression and protein-protein interactions to infer new transcription regulations. We take it as an example to illustrate how new TRs are discovered based on Hsf1 under the conditions of cell cycle and DNA Damaging. During the extensive search, the parameters are set as follows: *T*_start_=1, *T*_end_=0.01, *N* = 3000. After adding the extra members by stimulated annealing extensive searching, the Score(*L*) is 15.09 under Cell Cycle and Score(*L*) is 9.22 under DNA Damaging. If complexes are active under several conditions, we take their overlapping part in the extensive searching results. Figure 6 shows the topology of seed and final result of Hsf1 (YGL073W) and their expression profiles. We can note that the two sets of expression profiles exhibit a highly coherent similarity under the conditions of cell cycle and DNA Damaging, which also validates the scoring function. Based on this, we can infer that Hsf1 transcriptionally regulates the target genes YMR186W, YKL117W, and YOR027W as well, which are both covered in the extensive searching of Cell Cycle and DNA Damaging.

We validated our prediction results from three aspects: (1) we retrieved and compared with literature works which predicted the same TRs; (2) we detected the conserved binding motifs from the target genes in the seed and examined whether there were matches in the promoter of the predicted target; (3) we examined whether the function of predicted target genes was consistent with those of target genes in the seed. Of course, the final validation for the prediction result should depend on the biology experiment in the cell cycle condition. We found that the results by [32,33] and [34,35] supported our newly discovered TRs: Hsf1 regulates YMR186W and Hsf1 regulates YOR027W.

**Figure 7:**
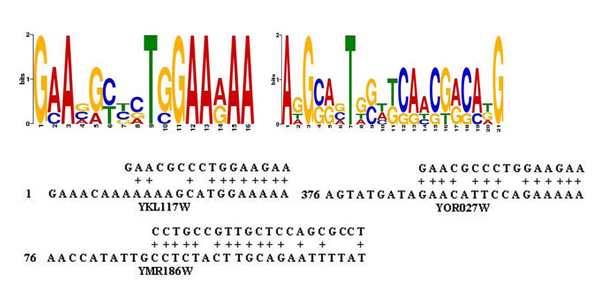
**Conserved motifs found in the seed and predicted target genes.** Two motifs predicted by MEME [36] from the upstream 600p of the five coding genes in the seed. The first motif is consistent with a known Consensus Motif (GAAXXTTCXXGAA) for Hsf1 in TRANSFAC.

However, we have not found direct evidence to support that Hsf1 regulates YKL117W. Maybe, it is a good idea to find evidence from binding motifs to support this. There are two significant binding motifs induced by the tool MEME from the upstream 600bp of the coding genes in the seed, which are shown in Figure 7. The first motif is consistent with a known Consensus Motif (GAAXXTTCXXGAA) for Hsf1. We found that there is a mach to the first motif in the 600bp upstream of YKL117W, YOR027W, and there is a match to the second motif in the upstream of YMR186W. Finally, we compared the function of Hsf1, the coding genes in the seed and the predicted target genes. SGD has an annotation for Hsf1 as following: ’Hsf1 regulates the transcription of hundreds of targets, including genes involved in protein folding, detoxification, energy generation, carbohydrate metabolism, and cell wall organization. Deletion of Hsf1 is lethal and mutants are defective in several processes including maintenance of cell wall integrity, spindle pole body duplication, protein transport, and cell cycle progression’. Meanwhile, we conducted a function enrichment analysis for the ten genes YLR216C, YLL026W, YPL240C, YAL005C, YLL024C, YDR214W, YNL007C, YMR186W, YKL117W, and YOR027W. One finding is that these genes have a common function of ’protein folding’, which belongs to the functional scope of Hsf1. 

For other extensive searches, in order to guarantee the accuracy, we only consider those complex seeds whose coherence perfectly consistent with the activity of their transcription factor in three conditions. Table 3 shows the results of newly predicted TRs and extra members for the perfect condition-dependent complex seeds listed in Table 2. The superscript number on the predicted target genes corresponds to that of their seed, which have the same TF.

**Table 3:** Predicted TRs(extra members) for perfect complex

Condition	TF	Predicted Target Genes
c2,c3	***CIN5 (YOR028C)***	YLR441C, YIL148W
c1	***YHP1 (YDR451C)***	YOL090W,YPL153C,YDR097C,YER095W, YMR078C,YNL312W,YMR200W,YPR080W, YJL173C
c1	***TOS4 (YLR183C)***	YML063W^1^,YGL048C^2^,YMR078C^3^, YOL012C^3^, YBR111W-A^3^
c1,c2,c3	***HAP3 (YBL021C)***	YEL024W, YJR121W, YGR183C
c1,c2,c3	***RNP4 (YDL020C)***	YDR394W, YOR261C, YIL075C, YMR276W
c1	***UME6 (YDR207C)***	YKL178C
c1,c2,c3	***ABF1 (YKL112W)***	YLR421C^1^,YFR052W^1^,YOL041C^2^, YER006W^2^, YGR103W^2^, YNL061W^2^, YER126C^2^, YMR128W^2^, YKL009W^2^, YDL150W^3^
c1,c3	***HAP4 (YKL109W)***	YKR065C, YCR012W
c1,c2	***HCM1 (YCR065W)***	YPL093W, YLR222C
c1,c2,c3	***DEP1 (YGL013C)***	YNL178W

## Discussion 

In this paper, we proposed a framework to discover conditional co-regulated protein complexes by integrating TR, GE and PPI data. This kind of protein complexes has three remarkable features: the coding genes of the member proteins share the same transcription factor, under certain condition the coding genes express co-ordinately and the member proteins interact mutually as a complex to implement a common biological function. Comparing to the existing works, one advantage is that our method not only uses the coding genes expression to measure the conditional protein activity but also takes the upstream TF activity into account to study their influence on protein complex. In the experiment, we observed some typical cases in which protein complexes’ coding gene coherent degree is strongly associated with their TF activity under different conditions. Another contribution is that we advanced the procedure of discovering co-regulated protein complex to discover potential unknown transcriptional regulation based on the tight relationships among co-regulation, co-expression and protein interaction. Because our work is based on the integration of several heterogeneous data sources, the result of the work could be influenced by the data quality in several aspects. The first one is the missing value in gene expression profiles. Besides the usual ways to directly assign value zero or the average value of the gene row to them, many other approaches have been proposed such as Singular Value Decomposition (SVD) based method (SVDimpute), weighted K-nearest neighbors (KNNimpute). Brock[37] has examined which imputation method is optimal for a given data set. Optimal imputation method should balance computation cost and untrue estimation. The second one is the available amount of the protein-protein interaction and transcriptional regulation data. Detecting them exactly is still a challenge in the field. In particular, it is hard to distinguish the false positive data. Another one is that an accurate prediction of TR under different conditions is very important for our work. Although this work focuses on the special kind of protein complex, it could help to understand the protein complexes’ organization and functional behaviour. Meanwhile new TRs are predicted based on the tight linkage between co-regulation, co-expression and protein-protein interactions. During the extensive search, it cannot detect all TRs for a species one time, but it provides an approach to exploit TRs from the complex mechanism. To make this method widely applicable, two real-life difficulties should be taken with caution. These include: (1) Time-course GE datasets with time points exceeding 10 for species except for yeast are not too many. In fact, most of them are knock-out experiments, which usually re-sample no more than 3 times. It is hard to measure the genes’ correlation with such few number of time points. (2) In this work, we used the Transcriptional regulation data directly. In fact much TR information could be extracted from other types of data like TFs binding. Meanwhile, the TF not only could act as promotion, but also repression, which will be considered in our future work. When the data become abundant and available, we believe our proposed method would be applicable for more species.

## Conclusions 

This study proposed the concept of conditional co-regulated protein complexes and developed a framework to discover them by integrating transcriptional regulation data, gene expression data, and protein-protein data. By linking these three types of data, the coherence change of the conditional co-regulated protein complexes influenced by the activity of TFs was observed, which implied that the functions of the proteins complexes were condition-dependent. We also reported newly inferred transcriptional regulations and validated the result rigorously.

## Competing interests

The authors declare that there are no competing interests.

## Authors' contributions

Luo and Liu initiated the main idea of the paper. Luo conducted all of the programming and computational experiments. Liu and Li supervised the whole work. The writing of the manuscript was conducted by Luo, and revised by Luo, Li and Liu.

## Supplementary Material

Additional file 1 The protein-protein interaction data is contained in protein protein interaction.mat. Click here for file

Additional file 2 Transcriptional regulation data is contained in transcriptional regulation.mat. Click here for file

Additional file 3 Gene expression data is contained in gene expression.mat. Click here for file
